# Cross-Platform Comparison of Amino Acid Metabolic Profiling in Three Model Organisms Used in Environmental Metabolomics

**DOI:** 10.3390/metabo13030402

**Published:** 2023-03-08

**Authors:** Jessica C. D’eon, Brian P. Lankadurai, André J. Simpson, Eric J. Reiner, David G. Poirier, Greg C. Vanlerberghe, Myrna J. Simpson

**Affiliations:** 1 Department of Chemistry, University of Toronto, 80 St. George Street, Toronto, ON M5S 3H6, Canada; 2 Environmental NMR Centre and Department of Physical and Environmental Sciences, University of Toronto Scarborough, 1265 Military Trail, Toronto, ON M1C 1A4, Canada; 3 Ontario Ministry of the Environment, Conservation and Parks, 125 Resources Road, Toronto, ON M9P 3V6, Canada; 4 Department of Biological Sciences, University of Toronto Scarborough, 1265 Military Trail, Toronto, ON M1C 1A4, Canada

**Keywords:** NMR spectroscopy, LC-MS/MS, *Daphnia magna*, *Eisenia fetida*, *Nicotiana tabacum*, metabolite profiling

## Abstract

Environmental metabolomics is a promising approach to study pollutant impacts to target organisms in both terrestrial and aquatic environments. To this end, both nuclear magnetic resonance (NMR)- and mass spectrometry (MS)-based methods are used to profile amino acids in different environmental metabolomic studies. However, these two methods have not been compared directly which is an important consideration for broader comparisons in the environmental metabolomics field. We compared the quantification of 18 amino acids in the tissue extracts of *Daphnia magna*, a common model organism used in both ecotoxicology and ecology, using both ^1^H NMR spectroscopy and liquid chromatography with tandem MS (LC-MS/MS). ^1^H NMR quantification of amino acids agreed with the LC-MS/MS quantification for 17 of 18 amino acids measured. We also tested both quantitative methods in a *D. magna* sub-lethal exposure study to copper and lithium. Again, both NMR and LC-MS/MS measurements showed agreement. We extended our analyses with extracts from the earthworm *Eisenia fetida* and the plant model *Nicotiana tabacum*. The concentrations of amino acids by both ^1^H NMR and LC-MS/MS, agreed and demonstrated the robustness of both techniques for quantitative metabolomics. These findings demonstrate the compatibility of these two analytical platforms for amino acid profiling in environmentally relevant model organisms and emphasizes that data from either method is robust for comparisons across studies to further build the knowledge base related to pollutant exposure impacts and toxic responses of diverse environmental organisms.

## 1. Introduction

Environmental metabolomics is a rapidly growing area of research that has contributed to novel findings in many various disciplines by providing rapid and holistic data about metabolic perturbations due to disease or some external stressor [1,2,3]. Environmental metabolomic studies focuses on environmental perturbations, such as pollutants and climate change, that impart stress on organisms dwelling in aquatic and terrestrial ecosystems [4]. Yet, environmental metabolomics faces unique challenges because of the diverse and large number of model organisms that can be studied and the lack of *a priori* knowledge about the metabolome, genome or proteome for environmentally-relevant organisms [4,5]. As such, non-targeted metabolomic studies using nuclear magnetic resonance (NMR) spectroscopy were more prevalent due to the ease in sample preparation and the ability of these techniques to yield information about a wide range of metabolites [5,6,7,8]. ^1^H NMR spectroscopy has been the primary tool with validation of metabolite structural assignments using two-dimensional (2-D) experiments and readily available database information [9,10,11,12]. In addition, ^1^H NMR spectroscopy is fully quantitative and highly reproducible [8,13]. Studies have reported that analysis of small molecules yield relative standard deviations of <1% [14,15]. NMR also has a large dynamic range which facilitates the analysis of complex mixtures with components of varying concentration [13,14,16].

More recently, the field of environmental metabolomics has grown to include sensitive mass spectrometric (MS) methods based on the knowledge gained from earlier NMR studies. For example, with the keystone water flea, *Daphnia magna*, the tiny size (high µg to low mg range, depending on age and life stage) necessitates that more than one organism be pooled for extraction prior to NMR analysis [12]. There are other techniques available to study the *D. magna* metabolome of individuals, such as in vivo NMR spectroscopy [17] however, these approaches are difficult to apply to large sets of organisms and with multiple environmental variables. Therefore, there is a need to use targeted MS/MS techniques for the quantification of endogenous metabolites in environmentally relevant organisms or samples of limited mass, such as fish mucus [18], in addition to metabolic profiling by NMR spectroscopy [19,20]. A particular group of metabolites of interest include free amino acids which are commonly used to assess changes in the metabolic profile of various organisms [4], including earthworms [21,22,23,24], *Daphnia* [25,26,27,28], and various fish [29,30,31,32,33,34,35] and plant species [10,36,37,38,39,40,41,42,43,44,45]. Amino acids are commonly analyzed because changes in their concentrations can be used to examine mechanisms such as oxidative stress, disruptions to energy metabolism and protein degradation. Consequently, amino acids are a key group of metabolites that can be used with other endogenous metabolites to form an understanding of the molecular-level perturbations due to various environmental stressors, such as sub-lethal pollutant exposure or nutritional stress [27,46].

**Figure 1:**
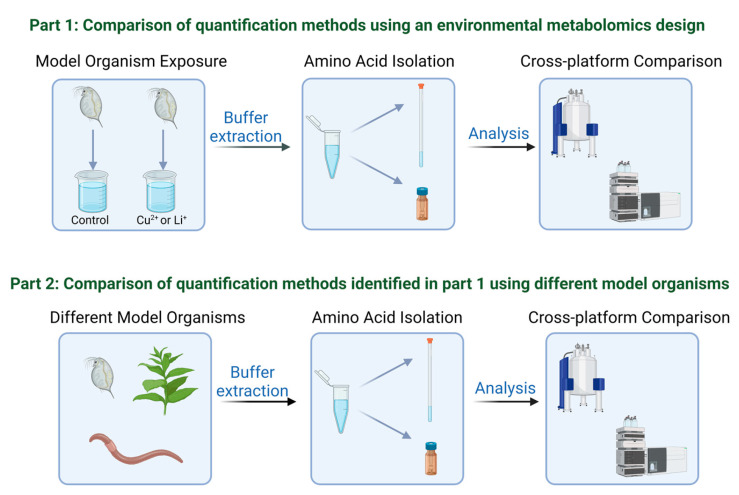
Schematic outlining the two part approach used in this study to compare amino acid quantification methods using nuclear magnet resonance (NMR) spectroscopy and liquid chromatography with tandem mass spectrometry (LC-MS/MS). Created with www.biorender.com.

Environmental metabolomic investigations provide critical information about molecular-level perturbations but one of the challenges related to this area of research is the shear number of model organisms that are used in addition to the use of different analytical platforms. There is a need to examine quantification of amino acids using different techniques, to determine the extent of compatibility across species and analytical approaches for improved standardization of workflows and results [2]. As such, the objective of this study was to compare the quantification of amino acids in three model organisms using both LC-MS/MS and ^1^H NMR spectroscopy on the same polar extracts. The model organisms included: *D. magna* which is a commonly used model in ecotoxicology and metabolomics [47], the earthworm, *Eisenia fetida*, a model organism for assessing soil pollution [4] and the well-studied tobacco plant, *Nicotiana tabacum* which is commonly used for assessing environmental variations on plant biochemistry [48,49,50,51,52]. The overall objective was to compare quantification of amino acids by ^1^H NMR and LC-MS/MS for environmentally relevant model organisms to determine if the two platforms agree and produce data that is transferable which is important for standardizing environmental metabolomic methods that use different approaches. This approached involved two main experiments (Figure 1) where we first examined different quantification methods using an environmental metabolomics exposure study with *D. magna* and then applied the approach to a comparison of amino acid profiling in three different model organisms (*D. magna*, *E. fetida*, and *N. tabacum*). This cross-platform comparison is also important for establishing baseline knowledge related to further development and use of metabolomic methods for broadly assessing environmental impacts to target organisms in different environmental compartments. Given the diversity of model organisms used in environmental metabolomics, a direct comparison of results is important for establishing standard approaches and data evaluation.

## 2. Materials and Methods

Our study included a two part experiment as outlined in Figure 1. Part 1 examines different quantification approaches for assessing amino acid concentrations after exposure to sub-lethal concentrations of copper and lithium. Based on the results of part 1, we applied the same cross-platform comparison to three different model organisms to more broadly assess the results between different samples.

### 2.1. Daphnia magna Amino Acid Extraction

*Daphnia magna* neonates (<24 h old) were isolated from the ongoing culture maintained at the Ontario Ministry of the Environment, Conservation and Parks as detailed in Nagato et al. [53]. Sub-lethal exposure to copper and lithium were carried out over 48 h. Neonates were exposed to either copper or lithium at 50% of the lethal concentration that results in mortality to 50% of the population (LC_50_). The LC_50_ values were determined to be 24.8 µg/L and 2300 µg/L for copper and lithium respectively and exposure concentrations for copper and lithium of 12.4 µg/L and 1150 µg/L were used (see Nagato et al., [53] for full details). Control (unexposed) neonates were also isolated for comparisons to metal-exposed *D. magna.*

After exposure, amino acids were extracted using a polar metabolite extraction previously optimized for NMR-based metabolomics that has high reproducibility and isolates a range of polar metabolites [12,53]. *D. magna* neonates (~10 mg, dry weight) were flash frozen in liquid nitrogen, lyophilized and extracted by adding 750 µL of a 0.2 M monobasic sodium phosphate buffer solution (NaH_2_PO_4_•2H_2_O; 99.3%; Fisher Chemicals; Ottawa, ON, Canada) with 0.1% *w/v* sodium azide (99.5%; Sigma Aldrich, Oakville, ON, Canada) as a preservative and 10 mg/L of sodium 2,2-dimethyl-2-silapentane-5-sulfonate (DSS; 97%, Sigma Aldrich) as an internal calibrant/standard for ^1^H NMR analysis. Buffer solution was made with D_2_O (99.9%, Cambridge Isotope Laboratories Inc., Tewksbury, MA, USA) and adjusted to a pD of 7.4 using NaOD (30% *w*/*w* in 99.5% D_2_O; Cambridge Isotope Laboratories Inc.). Samples were vortexed for 30 s and sonicated for 15 min. Samples were then centrifuged for 20 min at 14,000 rpm (~15,000 g) and the supernatant was decanted into a new 1.5 mL centrifuge tube. The centrifugation procedure was repeated once more to remove any remaining particulate material. 50 µL of the resulting extract was reserved for LC-MS/MS analysis and the remaining solution (700 µL) was transferred to a 5 mm High Throughput^plus^ NMR tube (Norell Inc., Morganton, NC, USA) for ^1^H NMR analysis. In total, eight replicate extractions were performed (*n* = 8) for *D. magna.*

### 2.2. Eisenia fetida and Nicotiana tabacum Amino Acid Extraction

Mature *E. fetida* earthworms were isolated from a culture where earthworms have been maintained for metabolomic experiments since 2006 [54]. Twelve mature *E. fetida* earthworms (50–70 mg per earthworm, dry weight), each with visible clitellum were individually flash frozen in liquid nitrogen, lyophilized, and homogenized in a 1.5 mL centrifuge tube using a 5 mm stainless steel spatula. Wild-type (WT) tobacco (*N. tabacum L.* cv Petit Havana SR1) was grown as described by Alber et al. [52]. Three whole leaves (10–20 mg per leaf, dry weight) from each of four mature plants were severed and immediately flash-frozen in liquid nitrogen. The frozen leaves were then ground into a fine powder with a liquid nitrogen cooled mortar and pestle and lyophilized. Because proteins may interfere with the use of an internal standard by ^1^H NMR spectroscopy, a modified method was used for isolating free amino acids which includes steps that result in protein precipitation. Lyophilized earthworm and tobacco samples were extracted by adding 400 µL of methanol and 85 µL of water and vortexing for 15 s [55]. Next, 200 µL water and 400 µL chloroform were then added and the samples vortexed for 1 min. The samples were then allowed to sit at 4 °C for 10 min, and then centrifuged for 10 min at 12,000 rpm (~11,000 g). The methanol:water layer was decanted and dried under a steady stream of nitrogen gas. The samples were then reconstituted in 750 µL of the same D_2_O phosphate buffer as described previously. Of this extract, 50 µL was reserved for LC-MS/MS analysis and the remaining extract (700 µL) was transferred to a 5 mm High Throughput^plus^ NMR tube (Norell Inc., Morganton, NC, USA) for ^1^H NMR analysis. For both *E. fetida* and *N. tabacum*, twelve replicate extractions were performed (*n* = 12).

### 2.3. Amino Acid Profiling via ^1^H NMR

Amino acid standards were purchased from BioShop (Burlington, ON, Canada) or Sigma-Aldrich (Oakville, ON, Canada) and were all >98.5% purity. Amino acid standards as well as a mixture of standards were prepared in the same D_2_O buffer (as described previously) and analyzed by ^1^H NMR spectroscopy. Amino acid solutions were analyzed using a BrukerBioSpin Avance III 500 MHz spectrometer (Ettlingen, Germany) equipped with a ^1^H-^19^F-^15^N-^13^C 5 mm Quadruple Resonance Inverse (QXI) probe fitted with an actively shielded Z gradient. ^1^H NMR experiments were performed using Presaturation Using Relaxation Gradients and Echoes (PURGE) water suppression [56] which was previously developed within our laboratory specifically for environmental metabolomics applications using NMR spectroscopy. Data were collected using 128 scans, and 64 K time domain points. Preliminary experiments were performed to measure the spin-lattice relaxation time (*T_1_*) of amino acid standards and samples to ensure that nuclei were fully relaxed before subsequent NMR pulses (the recycle delay was 5 × *T_1_*) [57]. All ^1^H NMR spectra were apodized through multiplication with an exponential decay corresponding to 0.3 Hz line broadening in the transformed spectrum, and a zero filling factor of 2. All spectra were manually phased and calibrated to the trimethylsilyl group of the DSS internal reference (δ = 0.00 ppm). Amino acid peaks in samples were identified in each spectrum by comparison with individually analyzed standards and compared to NMR spectra available from the Madison Metabolomics Consortium Database and published studies [9,12]. The NMR resonances used for quantification are listed in Table S1. These resonances did not overlap with ^1^H resonance from other amino acids or other compounds that may have been co-extracted from the model organisms and have been confirmed using 2-D NMR spectroscopy in *D. magna* [12] and *E. fetida* [11]. Integration of these specific amino acid resonances in ^1^H NMR spectra was performed using the multi-integration package of AMIX (version 3.8.4). The integrated resonance areas were then imported into Microsoft Excel (Microsoft Corporation, Redmond, WA, USA) and concentrations in the samples were calculated using an external calibration curve constructed from the integrated resonances of the standards. ^1^H NMR spectra of amino acid standards as compared to resonances found in sample extracts are shown in Figure S1. Internal standard quantification was also evaluated for *D. magna* extracts. The same integrated resonances for amino acids were used as with external standard quantification. Resonance areas were quantified based on the integrated area for the trimethylsilyl resonance of DSS (δ = 0.00 ppm).

### 2.4. Amino Acid Profiling via LC-MS/MS

Amino acid standards and samples were analyzed using a Sciex API 4000 QTrap mass spectrometer (Applied Biosystems/MDS Sciex, Concord, ON, Canada) coupled to an Agilent 1200 LC system equipped with an Ultra Aqueous C_18_ LC column (10 mm × 4.6 mm, 3 µm, Restek Corp.) which is designed for the separation of low retention compounds. The analysis involved a linear methanol:water gradient, with 0.15% formic acid added as a modifier to both solvents. The developed LC gradient consisted of: 0–1 min. hold at 5:95 methanol:water; 1–3 min. move to 20:80 methanol:water; 3–6 min. move to 40:60 methanol:water; 6–6.5 min. move to 95:5 methanol:water; 6.5–8 min. hold at 95:5 methanol:water and then return to initial conditions. All solvents were LC-MS grade purity (Sigma Aldrich, Oakville, ON, Canada). The LC flow rate was set at 0.5 mL/min and an injection volume of 10 µL was used. The LC column temperature was set at 40 °C. Electrospray ionization in positive ion mode and multiple reaction monitoring (MRM) was used based on transitions reported in the literature [58]. The ion source temperature was set to 750 °C. Table S2 lists the MRM ions used for detection and the retention times of each amino acid measured. Repeat injections (*n* = 3) for each amino acid standard yielded a relative standard deviation from 2.3–11% (Table S2). NMR D_2_O buffer extracts (50 µL) were diluted 100–500 times with LC-MS grade water (Sigma Aldrich, Oakville, ON, Canada). Amino acids were quantified by external calibration with standards, except glycine which was calibrated by internal calibration using ^13^C 2-glycine (Cambridge Isotope Laboratories, Inc., Tewksbury, MA, USA). Analyst (version 1.5.1) was used for peak integration and amino acid quantification.

### 2.5. Statistical Analysis and Comparison of Method Agreement

A *t*-test (two-tailed, equal variances) was used to compare the absolute concentrations of amino acids between the two methods to determine statistical differences at α = 0.05. A *t*-test (two-tailed, equal variances) was also used to compare the relative changes in the absolute concentrations of amino acids obtained by ^1^H NMR and LC-MS/MS in *D. magna* after copper and lithium exposure at sub-lethal concentrations.

We used methods recommended by Bland and Altman [59] to determine the extent of agreement between ^1^H NMR and LC-MS/MS methods. Measuring agreement between two independent measurements where the true value is not known can be established by plotting the difference of the mean measurements against the average measurement. The mean of the difference along with the standard deviation are also tabulated and plotted to establish a region of agreement (near the mean but not higher than the mean ± 2 × the standard deviation). This approach was applied to the *D. magna* concentrations measured by ^1^H NMR and LC-MS/MS using external standard quantification for both methods. For selected amino acids, we also performed regression analysis from all three model organisms which is an indicator of correlations between measurement [59].

## 3. Results

**Figure 2:**
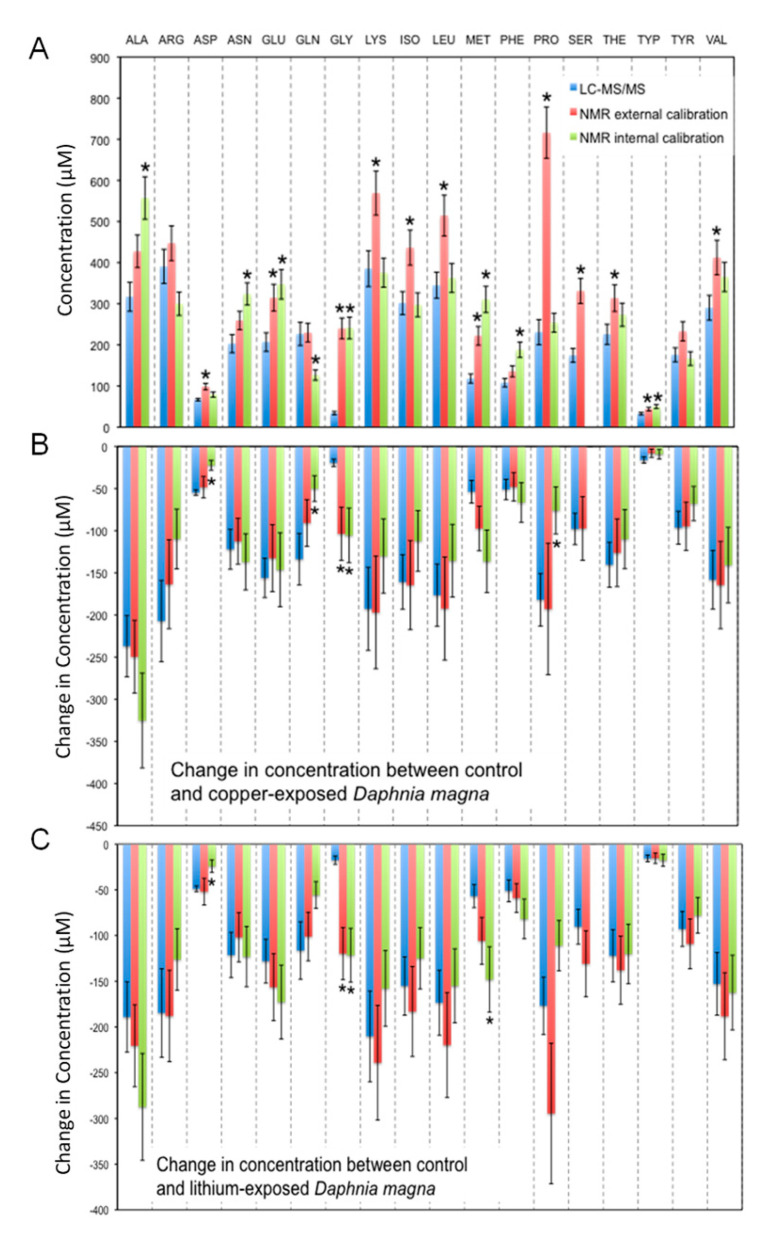
Concentrations of 18 amino acids observed in extracts of *Daphnia magna* by ^1^H NMR and LC-MS/MS (histidine was not quantified) expressed as (**A**) absolute concentrations observed in the extracts of control *D. magna*, or differences in amino acid concentrations observed between control *D. magna* and those exposed to (**B**) copper or (**C**) lithium. Any significant differences (α = 0.05) between measured concentrations are denoted with an asterisk (*). ALA = alanine, ARG = arginine, ASP = aspartate, ASN = asparagine, GLU = glutamate, GLN = glutamine, GLY = glycine, ILE = isoleucine, LEU = leucine, LYS = lysine, MET = methionine, PHE = phenylalanine, PRO = proline, SER = serine, THR = threonine, TRP = tryptophan, TYR = tyrosine, VAL = valine.

An overall comparison of the measured concentrations of amino acids in *D. magna* extracts is shown in Figure 2A. As a quantitative detector NMR has a large dynamic range, producing linear calibrations that span several orders of magnitude [14,16,60], whereas with MS, the dynamic range is analyte dependent and dependent on ionization and matrix effects [61,62]. Other considerations when examining quantification by NMR include the relaxation time (*T_1_*) of the sample components. Appropriate external and internal calibration methods require that the analytes of interest exhibit similar relaxation characteristics between the samples and standards and the results shown in Figure 2A demonstrates that this requirement was met for most amino acids analyzed. Histidine could not be quantified with certainty in these extracts by ^1^H NMR because the only proton resonances for histidine that did not overlap with other resonances were those on the carbons of the imidazole functional group at 8.03 ppm and 7.4 ppm (Figure S1). This would appear ideal as few other endogenous metabolites have resonances in this region. However, as the nitrogen atoms on the imidazole ring have pKa values around 6 [63] using a buffering system at pD 7.4 (~pH 7) can result in different charge states with very small changes in pH, which in turn causes the resonances for the C-H protons to shift slightly. The issue is confounded as the standards tended to shift downfield to 8.06 and 7.15 ppm, while the *D. magna* samples shifted upfield to 7.96 and 7.11 ppm. As evident from Figure 2A, there is a difference between ^1^H NMR results obtained using an internal (DSS) standard versus external standards for quantification. For some amino acids (Figure 2A), the concentrations determined using DSS alone were statistically different than those observed using external standard quantification by both ^1^H NMR and LC-MS/MS. But in other cases, both internal and external standard quantification methods for ^1^H NMR were similar with each other (e.g., glycine; Figure 2A).

**Figure 3:**
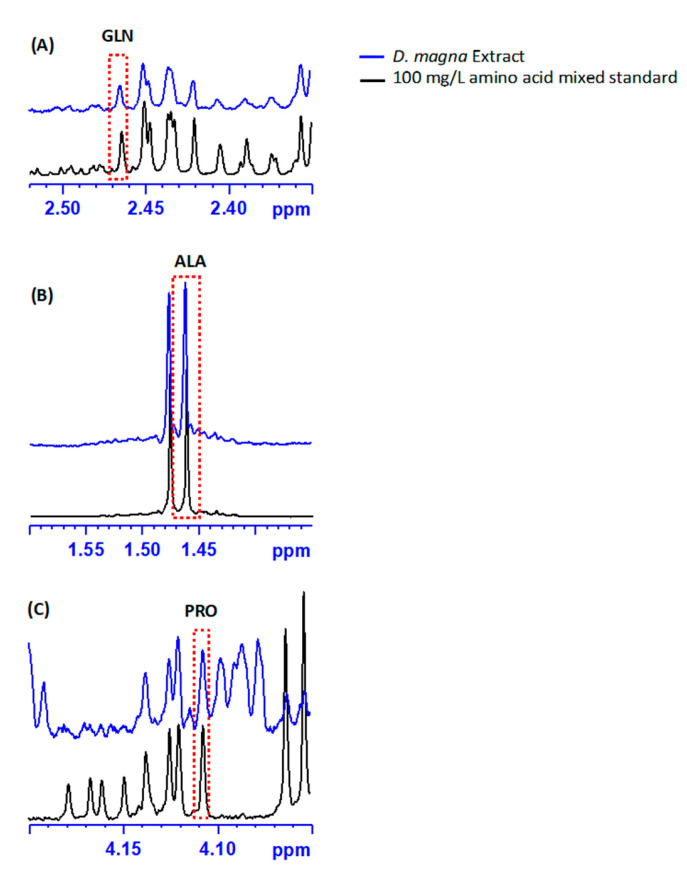
A comparison of the ^1^H NMR spectra of a *D. magna* extract and a mixed amino acid standard (100 μg/L), both in D_2_O buffer, highlighting the regions used for the quantification of (**A**) glutamine (GLN), (**B**) alanine (ALA), and (**C**) proline (PRO). A red dotted box indicates the specific resonance used for quantification for each amino acid.

This excellent agreement is likely due to the concentration of these amino acids in *D. magna* as well as a lack of matrix effects or other interferences. For example, the ^1^H NMR spectra of a *D. magna* extract is shown together with the ^1^H NMR spectra of a 100 μg/L mixed amino acid standard in Figure 3, where the *x*-axis is focused on the resonances used for quantification of glutamine, alanine and proline. Four unobstructed resonances for glutamine with no elevation or disturbance of the baseline between the samples and the standard are visible between 2.41 and 2.47 ppm in Figure 3A.

**Figure 4:**
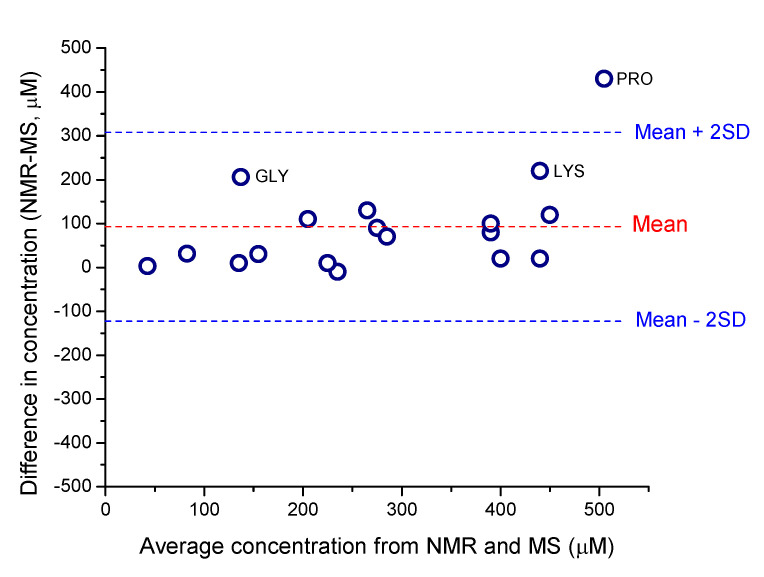
Evaluation of measurement agreement between ^1^H NMR and LC-MS/MS based on Bland and Altman [59]. The difference of the mean measurement plotted against the average measured concentration compares the agreement between the measurements within the calculated standard deviation (SD). Concentration data are listed in Table S3. GLY = glycine, LYS = lysine and PRO = proline.

To further evaluate the agreement between LC-MS/MS and ^1^H NMR spectroscopy measurements using external calibration of amino acids *D. magna* extracts, we compared the difference of the average measurements versus the average concentration of measurements (Figure 4), based on the method of Bland and Altman [59]. This method is employed when the true measurement value is not known and avoids bias when comparing two independent measurements [59]. Measurements are considered to be in good agreement if the difference in concentration between the measurements is within two standard deviations of their mean value [59]. By applying this method to evaluate agreement all amino acids except for proline fall within the agreement range (Figure 4). The difference between measurements (Figure 4; Table S3) for arginine, aspartate, glutamate, methionine, phenylalanine, tryptophan, tyrosine, and valine are closest to zero (mean differences ranged from -10 to 31 µM; Figure 4). The amino acids alanine, asparagine, glutamate, isoleucine, leucine, serine, and threonine exhibit greater average differences between measurements (Figure 4; Table S3) but are still within two standard deviations and considered to be in agreement [59]. For some of these amino acids, the underlying baseline is slightly elevated, suggesting small underlying interferences, as can be seen for alanine between 1.45 and 1.50 ppm in Figure 3B, and for the other amino acids (Figure S1). The overall similarity between sample and standard suggests no underlying interferences in the *D. magna* ^1^H NMR spectra for glutamine, which supports the excellent agreement observed (Figure 4; Table S3).

**Figure 5:**
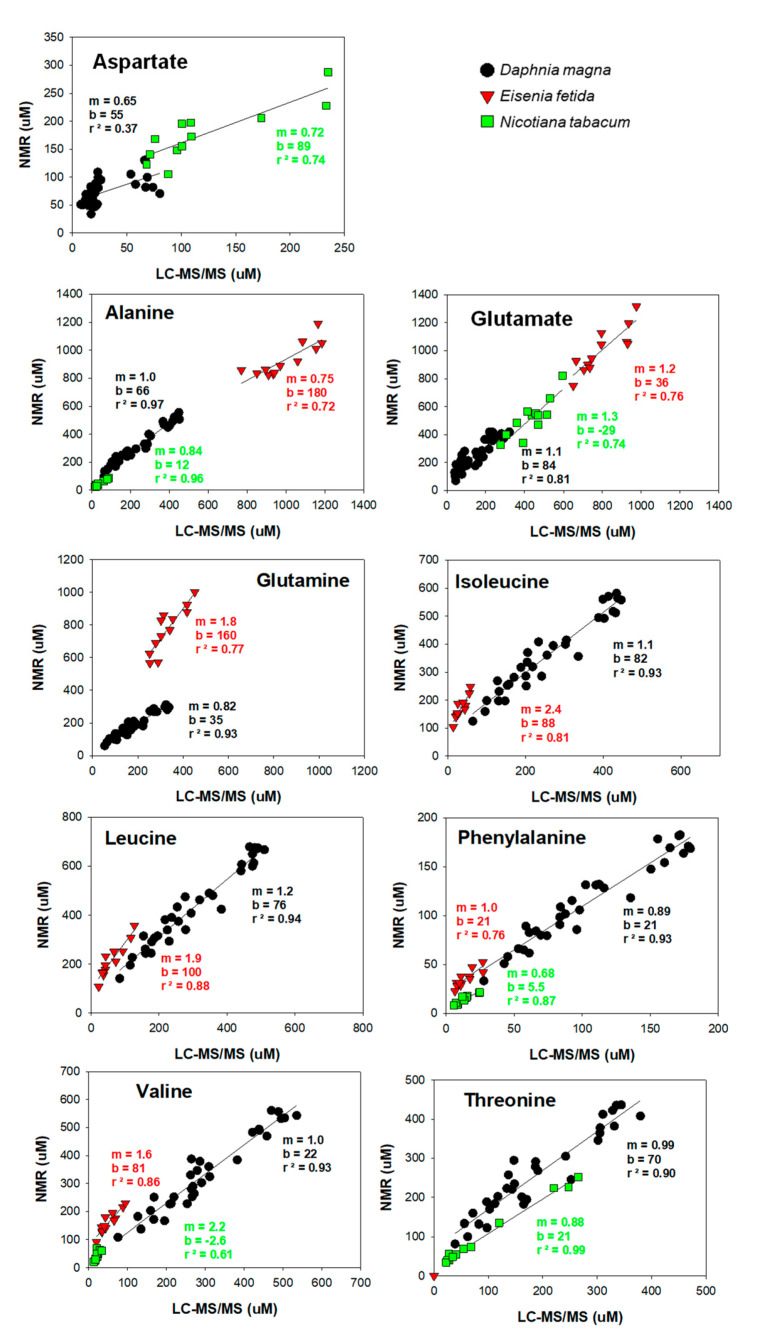
Comparison of amino acid quantification by ^1^H NMR and LC-MS/MS in polar extracts from three different environmentally relevant model organisms.

The focus of our study was on the quantification of amino acids found in *D. magna* for metabolomic studies. However, to explore if quantification by both LC-MS/MS and ^1^H NMR would yield the same results in extracts from other organism models, we also analyzed selected amino acids using external calibration methods by both ^1^H NMR and LC-MS/MS for *E. fetida* earthworms and the model plant *N. tabacum*. As mentioned previously, we observed some broadening of the NMR internal standard (DSS) so we did not proceed with internal quantification of amino acids in *E. fetida* and *N. tabacum* extracts. Overall, we found good agreement for detectable amino acids in all three organisms (Figure 5). The correlation coefficients (r^2^ values) are higher than 0.70 except for two correlations (aspartate in *D. manga* and valine in *N. tabacum*). This is likely due to the low concentrations of both amino acids which may have been below the limit of quantification. When amino acids were present above the limit of quantification, then the accuracy of detection improved, and we observed strong linear correlations between absolute concentrations measured by both ^1^H NMR and LC-MS/MS for all three model organisms (Figure 5).

## 4. Discussion

Overall, we generally found good agreement across platforms and with extracts from different organism models. We did observe some differences, depending on the approach, which will be discussed in detail in this section. Using an internal standard for quantification of ^1^H NMR spectroscopy data agreed less for *D. magna* extracts (Figure 2). There are several challenges with using internal standards for quantification in ^1^H NMR spectroscopy. The internal standard must not have overlapping resonances with compounds of interest which is why we chose DSS as it has an unobstructed resonance at δ = 0.00 ppm which also serves to calibrate the chemical shift. However, the internal standard may interact with other components in the sample matrix and this can result in changes in relaxation or line broadening of the internal standard. DSS is a commonly used internal calibrant because it is highly soluble in D_2_O and typically does not interfere with other compounds of interest. Sodium 3-trimethylsilyltetradeuteropropionate (TSP), is another NMR calibrant that is also used but both DSS and TSP can bind to proteins that may be present in sample extracts and this can result in broadening and/or loss of signal intensity [64,65]. Therefore, as an internal standard, this may result in an overestimation of metabolite concentrations. Also, such interactions may alter the *T_1_* value of the internal standard making quantification less accurate for some compounds. With *Daphnia* extracts, we did not observe broadening of DSS which suggests that internal calibration is a viable method for quantitative amino acid profiling. However, in other organisms, the co-extraction of peptides may limit the use of DSS as an internal standard. Interestingly, proline quantification was most variable of the amino acids (Figure 2A). This is likely due to differences in proline relaxation behavior when in a sample matrix as compared to a standard. To test this, we extended the delay time and observed that the concentration of proline decreased from 720 ± 60 µM to 560 ± 50 µM. This suggests that there is sample interference with proline that may limit the quantitative reliability in *Daphnia* extracts by ^1^H NMR analysis. We also noted that proline may have some interference due to the proximity to other resonances in the ^1^H NMR spectrum (Figure 3C). However, this would also manifest in the internal quantification results as well. As such, it is more likely that the relaxation of proline is variable and differs than the external standards. Further investigation is warranted to better understand the source of this variability and limitations associated with the absolute quantification of proline in *Daphnia* extracts.

We also found some differences in quantification when using different ^1^H resonances for integration. In the *D. magna* extracts the amino acid methionine gives three resonances that are not overlapped, the two downfield peaks of a triplet at 2.6 ppm (MET1 and MET2) and a singlet at 2.1 ppm (MET3) from the three protons of the methyl thioether (Figure S2). By LC-MS/MS the methionine concentration was found to be 140 ± 10 µM (Figure 2A). Using the three ^1^H NMR peaks individually the concentration of methionine by external NMR calibration were: 170 ± 20 µM (MET1, 21% difference), 190 ± 20 µM (MET2, 36% difference), and 170 ± 20 µM (MET3, 21% difference), all of which agree reasonably well with the LC-MS/MS data (140 ± 10 µM). By internal NMR calibration these same resonances yield concentrations of: 310 ± 30 µM (MET1, 120% difference), 180 ± 20 µM (MET2, 29% difference), and 1100 ± 100 µM (MET3, 690% difference), where only MET2 gives a comparable concentration. Reasons for these differences likely involve the relaxation properties of the protons in the molecules, particularly the significant over-estimation when using the resonance from the methyl thioether functional group. Furthermore, selecting one concentration for the internal standard may be challenging given the range of concentrations of amino acids found in these samples. However, with *Daphnia* extracts, this method provided good agreement overall. As indicated previously, DSS broadening due to interactions with proteins that are co-extracted with amino acids may reduce the reliability of internal standard methods. With *Daphnia*, we did not observe DSS broadening but with the other two extracts included in our study (*E. fetida* and N. *tabacum*), broadening was observed despite that we attempted to remove proteins prior to isolation of the free amino acids. As such, the remainder of our discussion will focus on comparisons made with external calibration. However, we note here that quantitative NMR techniques such as Electronic REference To access In vivo Concentrations (ERETIC), can be employed for internal standard quantification [66,67] by NMR that may alleviate issues we observed here. In our study, because our aim was to compare two specific analytical platforms, we focused more on quantification methods that would apply directly to LC-MS/MS and not only ^1^H NMR spectroscopy.

When comparing external quantification methods (Figure 4), we noted that some amino acids, alanine, asparagine, glutamate, isoleucine, leucine, serine, and threonine, exhibited more variability between methods (Figure 4). No obvious interferences are visible for asparagine; however the concentrations were low (Table S3) and the resulting NMR resonance intensity was lower than that observed for other amino acids (Figure S1). As such, the lower sensitivity of NMR may also contribute to some of the observed concentration differences however, amino acids with the lowest concentrations (arginine, aspartate, tryptophan; Table S3) exhibited very small differences and this suggests that differences in sensitivity between ^1^H NMR and LC-MS/MS did not limit accurate quantification. For asparagine, the proton resonances of interest are adjacent to a chiral center, resulting in increased splitting that drives the peaks of interest closer to the baseline, which may contribute to difficulties during spectral integration. Serine in *D. magna* extracts also has obvious interferences via NMR, which are manifested as an elevated baseline (Figure S1) which likely increased the difference between the measured concentrations.

Three amino acids show more deviation from the mean of the differences: glycine, lysine and proline, although only the difference in proline concentrations lies outside the agreement area (> mean ± 2 × Standard Deviation). As discussed previously, proline is likely overestimated by ^1^H NMR spectroscopy due to interference from another compound or compounds that are altering the relaxation and spectral baseline. As shown in Figure 3C, proline was particularly difficult to quantify in *D.* magna extracts because the only resonance that does not overlap resides around 4.1 ppm which is in a similar region to other amino acid protons. With glycine and lysine measurement, there may be instrument-specific reasons for the discrepancy between measurements. Lysine quantification via NMR may be altered due to the inference of another nearby resonance (Figure S1). Glycine concentrations were significantly higher by ^1^H NMR analysis in the *D. magna* extracts (Figure 2 and Figure 4; Table S3) by both external and internal techniques as compared to concentrations determined by LC-MS/MS (Figure 2A). The resonance used for glycine quantification (Figure S1; Table S1) does not show any overlap or baseline distortion. Also, there do not appear to be any resonances that may be attributed to other compounds. To investigate this disparity further, we included an internal standard for glycine during our LC-MS/MS analysis (^13^C 2-glycine) as we suspected that glycine was underestimated by external calibration due to ion suppression from matrix effects during electrospray ionization [61,68]. Ion suppression due to matrix effects is a common disadvantage of LC-MS/MS analyses of metabolomics samples [19,68]. However, inclusion of the mass labeled glycine (^13^C 2-glycine) did not significantly alter the concentration of glycine determined by LC-MS/MS (without internal standard the concentration of glycine in the control *D. magna* D_2_O extracts was 30 ± 4 µM, with internal standard this increased to 34 ± 4 µM). Glycine has a retention time that is similar to other amino acids (Table S2) and this may also impact ESI efficiency. Interference and/or suppression can occur even with a mass-labelled internal standard and given that the transition ions for glycine are common (76 > 30 *m/z*), it is likely that LC-MS/MS may be underestimating glycine concentrations in *Daphnia* extracts. With ESI, the MS responses may vary with analyte concentration because of varying ionization efficiencies and varying ion suppression [69]. Another possible explanation for the high concentrations of glycine observed by ^1^H NMR is that, as with histidine, there are several common glycyl dipeptides [70], and it is possible that the protons from these small molecules are obstructing or overlapping with the NMR resonance for free glycine leading to the observed overestimation of the concentrations. However, 2-D NMR analysis of *D. magna* polar extracts did not reveal any overlap between the resonance used for glycine (δ = 3.55 ppm) quantification [12]. Furthermore, both internal and external quantification using ^1^H NMR show good agreement (Figure 2). As such, the observed discrepancy in concentrations determined for glycine by ^1^H NMR and LC-MS/MS warrants further investigation but is likely specific to sample matrix effects specific to *D. magna* extracts.

In environmental metabolomic studies, a common objective is to examine how metabolite concentrations fluctuate with an external stressor, such as an environmental pollutant. However, typically, only the fold change is reported (change in metabolite concentration relative to the control). This is mostly due to the vast amount of data generated in metabolomic studies and brevity in presenting such data. To investigate the use of relative changes in concentration for metabolomic data, we analyzed *D. magna* extracts from a companion study where *D. magna* were exposed to sub-lethal concentrations of copper, lithium and arsenic for 48 h [53]. Nagato et al. [53] reported a significant decrease in the concentration of all the amino acids in lithium or copper exposed *D. magna* relative to the controls (unexposed), whereas the amino acids were unchanged or increased in the arsenic exposures. These changes in the amino acid concentrations were attributed to a specific reaction of the *D. magna* to metal exposure, potentially via the incorporation of these amino acids into enzymes in response to the metal-induced stress, or it may represent changes in growth patterns between treatments. Figure 2B,C show the change in amino acid concentration observed between the copper and lithium exposure groups compared to control as determined by LC-MS/MS, and both external and internal standard NMR quantification. As with the *D. magna* extracts (Figure 2A), we observed agreement between the LC-MS/MS and NMR quantification. Again, internal standard use by NMR is significantly different for some amino acids. The only amino acid that has significantly different concentrations between the two techniques is glycine, which as described previously remains a topic of investigation.

One explanation for why there is better agreement between LC-MS/MS and ^1^H NMR when looking at concentration changes between groups of organisms as opposed to absolute concentrations is that any underlying ^1^H NMR baseline disruption is nullified. This hypothesis is visualized in plots of concentration by ^1^H NMR (external calibration) versus concentration by LC-MS/MS in Figure 5. The slope (m) for most of the amino acids, especially for the *D. magna* samples, is close to unity, which reflects the ability of ^1^H NMR to characterize differences in amino acid concentrations between samples. However, the values for the y-intercepts (b) are large, which may reflect underlying interferences that do not scale with changes in the amino acid concentration as well as differences in the detection limits of both analytical platforms. For example, threonine has a slope of 0.99, but an intercept of 70 µM. The concentration of threonine observed in the control *D. magna* was 250 ± 20 µM by LC-MS/MS and 320 ± 30 µM by ^1^H NMR, a difference of 70 µM which is similar to the y-intercept. However, this difference decreased when comparing the change in concentration between the control and copper-exposed animals, which was 150 ± 30 µM by LC-MS/MS and 130 ± 40 µM by NMR. The underlying interference, which is reflected in the elevated y-intercept, is almost entirely cancelled out when considering differences in concentration between two sample sets.

This analysis of the comparison of the changes in the amino acid concentrations between NMR and LC-MS/MS methods provides justification for the utility of NMR quantification in applications, such as metabolomics, where relative concentrations between related sample sets are more relevant than absolute concentrations. Furthermore, these observations highlight the excellent agreement between LC-MS/MS and ^1^H NMR quantification methods and demonstrate that these analytical platforms enable the same result. This is important as researchers often select one platform over the other and rarely use both in tandem [19]. Both techniques have advantages and disadvantages but when data acquisition is optimized, we found that the data are in excellent agreement with each other and for the most part, data are transferable from one platform to the other. These results are highly promising and demonstrate that both analytical platforms are robust and capable of quantifying amino acids in a variety of environmental samples. We note that we did not optimize the extraction methods, especially for *N. tabacum*, so the method of amino acid extraction used in our study may require further optimization for metabolomic studies with *N. tabacum.* Nonetheless, of the amino acids that were quantified, we report excellent agreement using both LC-MS/MS and NMR and demonstrate excellent promise for future studies which aim to quantify amino acids in these model organisms.

## 5. Conclusions

Overall, our study showed that ^1^H NMR quantification of amino acids generally agreed with LC-MS/MS quantification except for proline in *D. magna* extracts only. We also confirmed that accurate quantification of amino acids by both methods is dependent on the complexity of the biological matrix and the concentration of the amino acids in the extracts. *D. magna* extracts had the simplest biological matrix and the highest concentration of amino acids per mass biomass extracted, where both techniques agreed for all amino acids studied except for proline. The more complex *E. fetida* and *N. tabacum* extracts, which had a lower concentration of amino acids per biomass extracted, enabled the quantification of only selected amino acids. Our study confirmed that ^1^H NMR quantification via external calibration is an easy and reliable method to quantify metabolites present in extracts from environmentally relevant organisms. We also illustrated that both analytical platforms detect metabolic shifts similarly when used to quantify amino acids in an environmental metabolomics study of *D. magna* exposure to two metal pollutants. Because small organisms, such as *D. magna,* need to be pooled for ^1^H NMR-based metabolomics, the higher sensitivity afforded by LC-MS/MS is advantageous. We demonstrate that there is agreement in amino acid profiling by LC-MS/MS and ^1^H NMR and data collected by both methods are directly comparable. Our results are significant because it demonstrates that the direct translation of data across different model organisms and analytical platforms with certainty. This is critical for the future advancement of the use of metabolomics in environmental toxicology and biomonitoring programs, especially given the diversity of organisms and available analytical platforms for such investigations. Our work provides confidence in the measurement of endogenous metabolites across platforms and demonstrate the ubiquity of metabolomic investigations for ascertaining molecular-level perturbations with environmental stressors.

## Data Availability

All data are available in the main article or in the supporting materials. Raw data files (instrument files) can be obtained upon request from the corresponding author because of the privacy.

## Supplementary Materials

The following supporting information can be downloaded at: https://www.mdpi.com/article/10.3390/metabo13030402/s1, Figure S1: Comparison of the ^1^H NMR spectra of amino acid standards and of the tissue extracts of *Daphnia magna*, the earthworm *Eisenia fetida*, and the tobacco species *Nicotiana tabacum.* Resonances used for quantification are highlighted by a dashed box and also listed in Table S1.; Figure S2: ^1^H NMR spectra of a *D. magna* extract, a 100 μg/L individual methionine standard solution in D_2_O buffer and a mixture of amino acids (100 μg/L) in D_2_O buffer, highlighting the section highlighting the resonances that are attributed to methionine.; Table S1: The ^1^H NMR regions used for quantification of amino acids. Resonances are based on metabolic profiling using both ^1^H and two-dimensional NMR spectroscopy for *D. magna* extracts; Table S2: LC-MS/MS multiple reaction monitoring (MRM) mass transitions and relative standard deviation (%) of triplicate injections of amino acid standards.; Table S3: Comparison of amino acid concentrations measured using LC-MS/MS and ^1^H NMR spectroscopy on *Daphnia magna* D_2_O buffer extracts. Values are based on external standard quantification and expressed as averages (*n* = 8) with associated standard errors.
