# Supplementary material for: Cross-Platform Comparison of Amino Acid Metabolic Profiling in Three Model Organisms Used in Environmental Metabolomics
Source: Metabolites. 2023 Mar 8;13(3):402. doi: 10.3390/metabo13030402 (PMC10058405; doi:10.3390/metabo13030402)
Supplement: Supplementary file 1 [file metabolites-13-00402-s001.zip › metabolites-2259726-supplementary.pdf]

-Supplementary Materials-

Cross-platform comparison of amino acid metabolic profiling in three model organisms used in environmental metabolomics

Jessica C. D'eon[1a], Brian P. Lankadurai[1a], André J. Simpson[1], Eric J. Reiner[2], David G. Poirier[2], Greg C. Vanlerberghe[3], and Myrna J. Simpson[1*]

[1]Department of Chemistry, University of Toronto, 80 St. George Street, Toronto, ON, M5S 3H6. Environmental NMR Centre and Department of Physical and Environmental Sciences, University of Toronto Scarborough, 1265 Military Trail, Toronto, ON, M1C 1A4.

[2]Ontario Ministry of the Environment, Conservation and Parks, 125 Resources Road, Toronto, ON, M9P 3V6.

[3]Department of Biological Sciences, University of Toronto Scarborough, 1265 Military Trail, Toronto, ON, M1C 1A4.

[a]These authors contributed equally to this work.

*Corresponding author: Tel. (416) 287-7234, Fax: (416) 287-7279, E-mail address: myrna.simpson@utoronto.ca

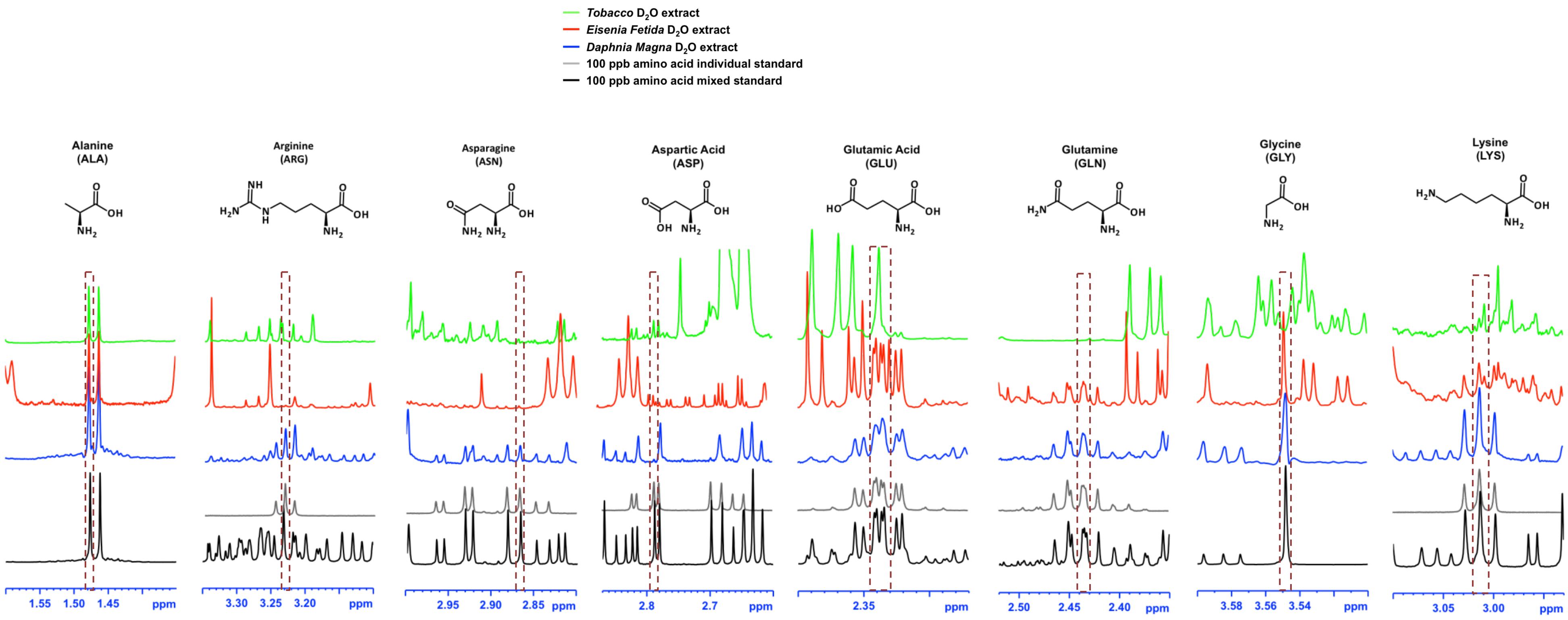

Figure S1: Comparison of the ^{1}H NMR spectra of amino acid standards and of the tissue extracts of *Daphnia magna*, the earthworm *Eisenia fetida*, and the tobacco species *Nicotiana tabacum*. Resonances used for quantification are highlighted by a dashed box and also listed in Table S1.

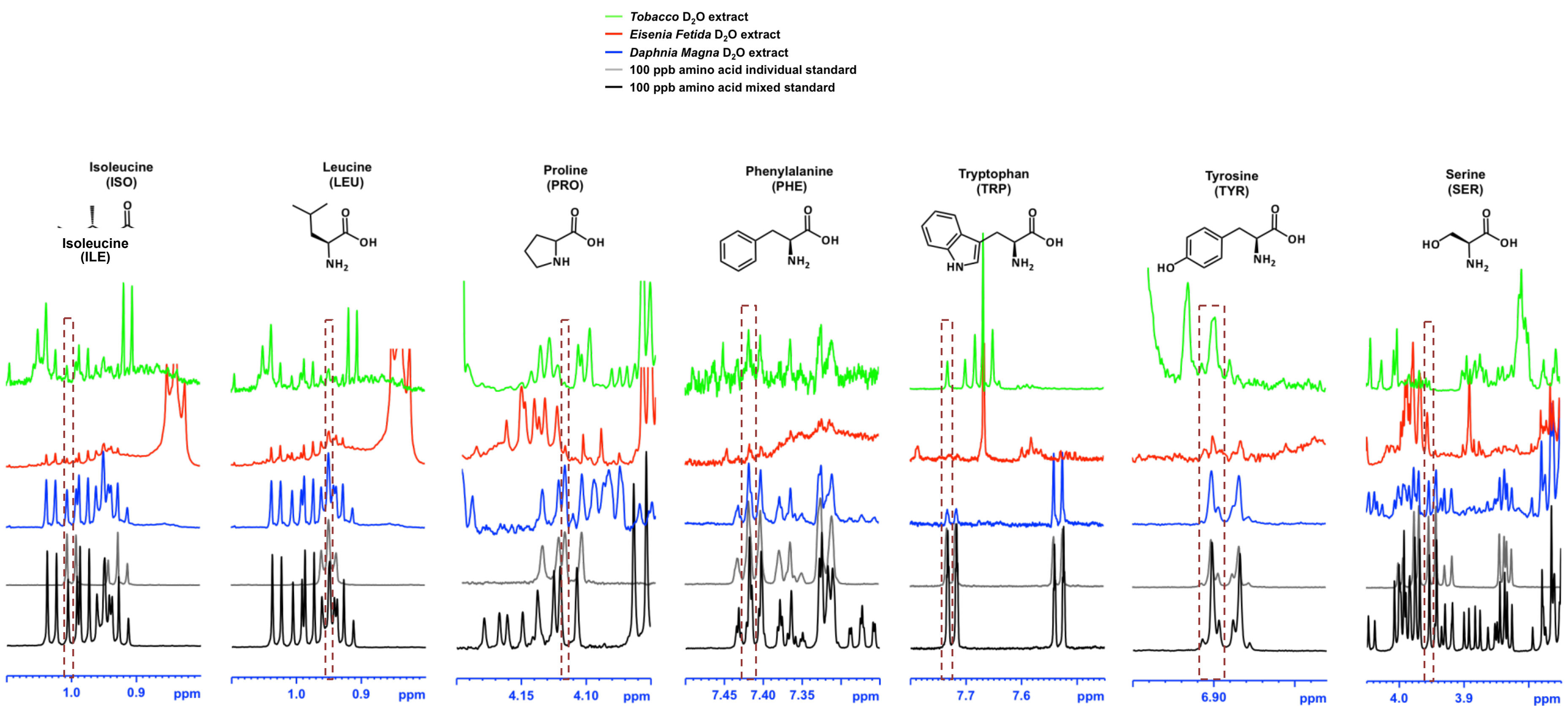

Figure S1 (continued): Comparison of the ^{1}H NMR spectra of amino acid standards and of the tissue extracts of *Daphnia magna*, the earthworm *Eisenia fetida*, and the tobacco species *Nicotiana tabacum.* Resonances used for quantification are highlighted by a dashed box and also listed in Table S1.

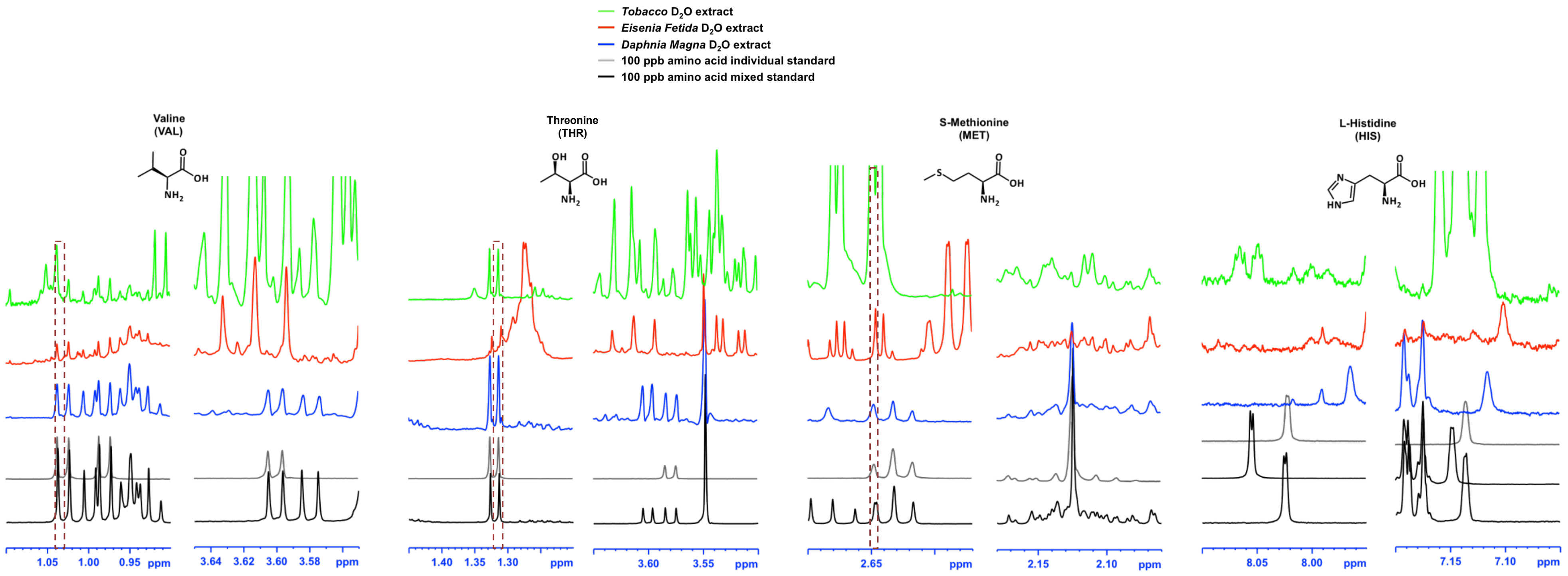

Figure S1 (continued): Comparison of the ^{1}H NMR spectra of amino acid standards and of the tissue extracts of *Daphnia magna*, the earthworm *Eisenia fetida*, and the tobacco species *Nicotiana tabacum*. Resonances used for quantification are highlighted by a dashed box and also listed in Table S1.

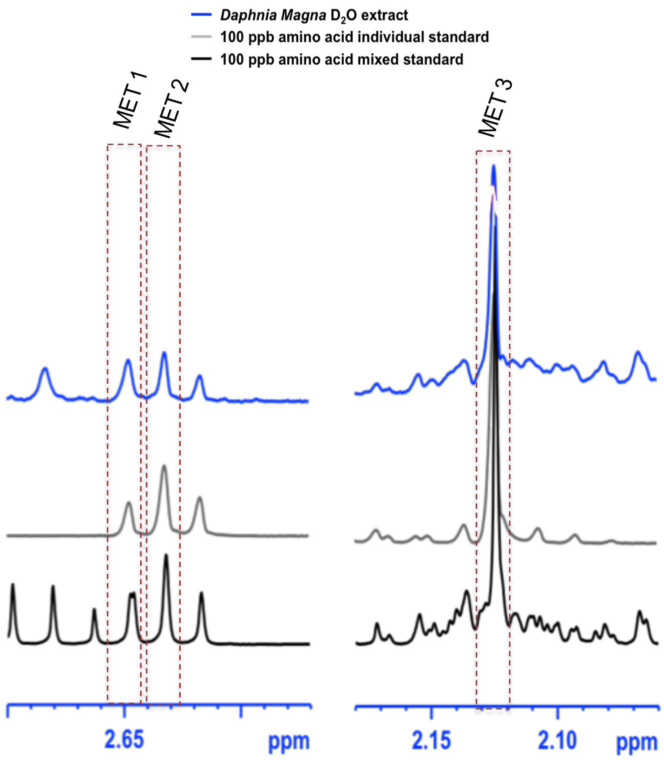

Figure S2: ^{1}H NMR spectra of a *D. magna* extract, a 100 µg/L individual methionine standard solution in D_2O buffer and a mixture of amino acids (100 µg/L) in D_2O buffer, highlighting the section highlighting the resonances that are attributed to methionine.

Table S1: The ^1H NMR regions used for quantification of amino acids. Resonances are based on metabolic profiling using both ^1H and two-dimensional NMR spectroscopy for *D. magna* extracts [1].

**Amino Acid**	**Chemical Shift Range (ppm)**	
	Left	Right
Alanine	1.482	1.4684
Arginine	3.2388	3.225
Aspartate	2.7941	2.784
Asparagine	2.8739	2.86
Glutamate	2.3466	2.3297
Glutamine	2.4429	2.4276
Glycine	3.5538	3.5443
Histidine	nd[a]	nd[a]
Isoleucine	1.0094	1.0023
Leucine	0.9551	0.9449
Lysine	3.0216	3.0048
Methionine	2.6514	2.6432
Phenylalanine	7.4252	7.4102
Proline	4.1227	4.1173
Serine	3.9564	3.9493
Threonine	1.3173	1.3102
Tryptophan	7.7414	7.255
Tyrosine	6.9118	6.8912
Valine	1.0432	1.0338

[a]nd = not detected (HIS was not detected in *Daphnia magna* extracts via ^1H NMR analysis; please see Results & Discussion section for more detail).

Table S2: LC-MS/MS multiple reaction monitoring (MRM) mass transitions and relative standard deviation (%) of triplicate injections of amino acid standards.

Amino Acid Standard	MRM Precursor ion > product ion (*m/z*)	t_R (min)	Triplicate Injection Relative Standard Deviation (%)
Alanine	89.9 > 44	2.2	5.0
Arginine	175 > 70.1<br>175 > 116.1	1.9	5.6
Aspartate	133 > 87	2.1	3.5
Asparagine	134 > 74	2.1	4.9
Glutamate	148 > 84.1<br>148.1 > 130	2.2	4.9
Glutamine	147.1 > 130.1<br>147.1 > 101.1	2.2	4.8
Glycine	76 > 30	2.0	13
Histidine	156.1 > 110<br>156.1 > 83	1.9	4.0
Isoleucine	132 > 69.1<br>132 > 86.1	4.4	3.2
Leucine	132 > 86.1	4.5	3.2
Lysine	147 > 84.1<br>147 > 130.1	1.8	3.6
Methionine	150 > 104<br>150 > 133	3.7	2.9
Phenylalanine	166.1 > 120<br>166.1 > 149.1	5.5	2.7
Proline	116 > 70.1	2.75	2.9
Serine	106 > 60	2.1	4.9
Threonine	120 > 74.1<br>120 > 56.2	2.2	3.7
Tryptophan	205.1 > 188.1<br>205.1 > 146.1	6.2	2.3
Tyrosine	182 > 136.2<br>182 > 165.1	4.4	2.3
Valine	118.1 > 72.1<br>118.1 > 55	3.2	4.4

Table S3: Comparison of amino acid concentrations measured using LC-MS/MS and ^{1}H NMR spectroscopy on *Daphnia magna* D_2O buffer extracts. Values are based on external standard quantification and expressed as averages (n=8) with associated standard errors.

**Amino acid**	**LC-MS/MS (µM)**	**NMR (µM)**	**Difference in measurement between NMR and MS (µM)**
Alanine	350 ± 60	430 ± 40	80
Arginine	430 ± 40	450 ± 40	20
Aspartate	67 ± 3	98 ± 8	31
Asparagine	150 ± 20	260 ± 20	110
Glutamate	230 ± 20	320 ± 30	90
Glutamine	240 ± 20	230 ± 20	-10
Glycine	34 ± 4[a]	240 ± 30[a]	206
Histidine	58 ± 7	nd[b]	na[c]
Isoleucine	340 ± 30	440 ± 40	100
Leucine	390 ± 40	510 ± 50	120
Lysine	330 ± 30	550 ± 50	220
Methionine	140 ± 10	170 ± 20	30
Phenylalanine	130 ± 10	140 ± 10	10
Proline	290 ± 40[d]	720 ± 60[d]	430
Serine	200±20	330 ± 30	130
Threonine	250 ± 20	320 ± 30	70
Tryptophan	41 ± 4	44 ± 4	3
Tyrosine	220 ± 20	230 ± 20	10
Valine	390 ± 40	410 ± 40	20

[a] Glycine quantification is likely underestimated by LC-MS/MS (see Results & Discussion section for more detail).
[b]nd = not detected in *Daphnia magna* extracts by ^{1}H NMR.
[c]na = not applicable because HIS was not quantified by NMR (see Results & Discussion section for more detail).
[d]Proline quantification was likely hindered due to the sample matrix (see Results & Discussion section for more detail).

References

1. Nagato, E.G.; Lankadurai, B.P.; Soong, R.; Simpson, A.J.; Simpson, M.J. Development of an NMR Microprobe Procedure for High-Throughput Environmental Metabolomics of *Daphnia magna*. *Magn. Reson. Chem.* **2015**, *53*, 745–753, doi:10.1002/mrc.4236.
